# Optimum periodicity of repeated contractile actions applied in mass transport

**DOI:** 10.1038/srep07800

**Published:** 2015-01-27

**Authors:** Sungsook Ahn, Sang Joon Lee

**Affiliations:** 1 Biofluid and Biomimic Research Center, Pohang University of Science and Technology, Pohang, 790–784, Korea; 2 Department of Mechanical Engineering, Pohang University of Science and Technology, Pohang, 790–784, Korea

## Abstract

Dynamically repeated periodic patterns are abundant in natural and artificial systems, such as tides, heart beats, stock prices, and the like. The characteristic repeatability and periodicity are expected to be optimized in effective system-specific functions. In this study, such optimum periodicity is experimentally evaluated in terms of effective mass transport using one-valve and multi-valve systems working in contractile fluid flows. A set of nanoscale gating functions is utilized, operating in nanocomposite networks through which permeates selectively pass under characteristic contractile actions. Optimized contractile periodicity exists for effective energy impartment to flow in a one-valve system. In the sequential contractile actions for a multi-valve system, synchronization with the fluid flow is critical for effective mass transport. This study provides fundamental understanding on the various repeated periodic patterns and dynamic repeatability occurring in nature and mechanical systems, which are useful for broad applications.

Periodically repeated patterns are pervasive in natural and artificial systems where characteristic spatial or temporal information can be determined. Gravitational forces cause tides in the ocean and Earth with diurnal and semidiurnal periods^1^. Earthquakes in a specific region are anticipated by repeated oscillation patterns^2^. Among the biological periodic processes, the rhythmic process is a central function of life, such as limb motions in walking, heart beating, metabolism, growth, hormone regulation, and circadian clocks^3^,^4^. In this context, pulsatile pressure and dynamic flow in arteries have attracted increasing interest to explain disease-related hemodynamic phenomena^5^.

To investigate the physiological dynamic phenomena occurring in nature, *in vitro* model systems have been usefully employed. In many model systems, the water-based fluid flows through rigid pipes have been handled. However, these studies have technical limitation in their plausible adoptability for actual dynamics. Selective mass transport with controllable flexibility^6^ thus has a great advantage in mimicking the real dynamics actually occurring in many biological systems. For example, human skin, a typical natural responsive membrane, acts as a permeable multifunctional membrane which interacts and responds to the surrounding environments such as light, heat, cold, humidity, chemicals, and mechanical stress^7^. Responsive materials have a great advantages in broad application areas such as controlled-release agents^8^,^9^, responsive coatings^10^ and artificial organs^11^. In addition to the molecular separation by size and charge for which traditional membranes are employed, responsive membranes are designed to dynamically respond to the varying environments. Although this dynamic aspect is essential in the behaviors of responsive materials, it has not been thoroughly investigated because suitable model systems are lacking.

Unlike energy transfer mechanisms (e.g., tides and earthquakes) that employ Fourier's law, mass transport is mainly governed by conventional physical laws including acceleration and gravitational rules. In this study, dynamic mass transport through stimuli responsive soft condensed matters (nanocomposites) is investigated as a new model system to study the dynamically repeated patterns occurring in contractile fluid flows. By changing the dynamic factors, the output patterns of permeate molecules are systematically modulated. Based on the obtained results, we propose a certain optimized pattern for periodic control in dynamic mass transport. This study is the first systematic investigation in which periodic repeatability and effective mass transport are conceptually combined. The obtained results would be useful for understanding various periodic patterns and dynamic mechanics which are abundant in various natural and artificial systems.

## Results

### Temperature-responsive nanocomposite

**Figure 1:**
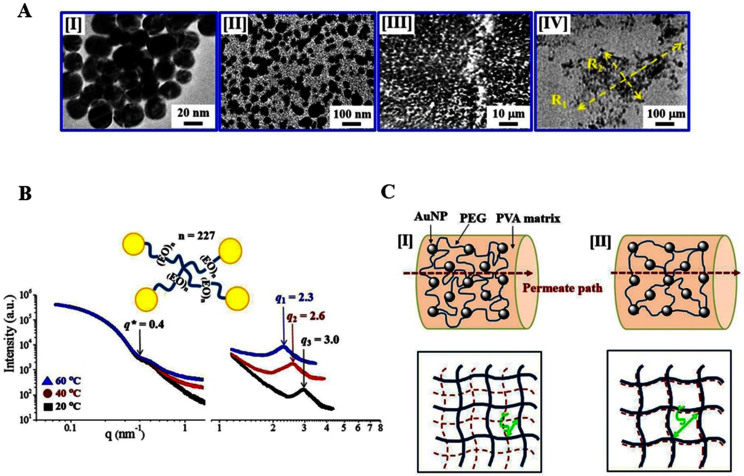
(a) [I] TEM image of gold nanoparticle (AuNP) use for linterlinking. Binary functional polyethylene oxide (PEO) having EO unit number of n = 227 (2PEG 10000) is used for linking AuNPs. Representative images of the designed nanocomposites. [II] TEM, [III] X-ray nanoscopy (XN) and [IV] X-ray microscopy (XM). (b) Small angle X-ray scattering (SAXS) results of the designed AuNP-PEG nanocomposite in solution state in broad *q* ranges (two Sample-to-detector distance conditions are combined). A critical *q* value (marked by q*) exist from which temperature-responsiveness is diversified, indicating dual regions of the designed network: stable large-scale domain at low *q* and responsive small-scale domain at high *q*. Characteristic size is determined according to the temperature detected by *q*_1_ (60°C), *q*_2_ (40°C) and *q*_3_ (20°C). (**c**) Illustration of responsive network structures. Pore size variation of the nanocomposites in PVA matrix induced by the stimuli-responsive PEOs through which permeates are transported. [I] Swollen PEOs generates (or block) small path for permeate molecules. [II] Shrunken PEOs allow relatively wide pathway for effective permeate transport. The correlation lengths (ζ) are determined based on the pictures below. Bold lines denote stable connection, whereas dotted lines indicates flexibly responsive chains by the external stimuli.

Citrate-stabilized colloidal gold nanoparticles (AuNPs) are prepared in aqueous solution. The concentration is adjusted to be around 2.4 × 10^12^ AuNPs/mL (Supporting Information)^12^,^13^. The transmission electron microscopy (TEM) mage in Figure 1a[I] confirms the average diameter of the prepared single AuNP is 20 nm diameter. Binary thiol end-capped functional polyethylene oxide (PEO) are incorporated for AuNP interconnection. The molecular weight between the junctions is adjusted to be M_p_ = 10,000 ((EO)_n_, n = 227). The TEM image in Figure 1a[II] shows that the average size of the clusters in nanometer-scale^14^,^15^. Considering the multi-reactive sites on a single AuNP, the number of incorporated PEO molecules is controlled to ×10, ×50, and ×100 times that of AuNPs (Supporting Information). A network is hardly formed if binary PEO monomers are only activated. However, highly interconnected composite clusters are formed as a result of multiple reactions of AuNP surface to thiol groups at the ends of the PEO. Multiple PEO linkages are attached to the surface of an AuNP to form networked assemblies. They also possess additional levels of complexity and anisotropy that can be exploited in self-assembly. The crosslink density (ρ) of the fully-linked network is inversely proportional to the molecular weight between the junction points (M_p_).

Nano-scale images are obtained at the 7C X-ray nanoimaging (XNI) beamline at Pohang Accelerator Laboratory (PAL, Pohang, Korea) using high X-ray absorption coefficient of Au^13^ (Supporting Information) (Figure 1a[III]). Beam size is adjusted to 100 μm × 100 μm at 6.7 keV energy. Spatial resolution is approximately 100 nm. A texture of a composite cluster is prominent as black dots because of embedded AuNPs of high X-ray absorption. In Figure 1a[IV], the X-ray micro imaging results obtained at the 6D X-ray Micro Imaging (XMI) beamline at PAL provide a larger field-of-view than XNI (Supporting Information). Its spatial resolution is approximately 4 μm at the sample-to-detector distance of 30 cm and FOV is adjusted to 1200 μm × 900 μm. Discrete clusters are detected by XMI and the size of the cluster is evaluated. Assuming that the cluster is in an ellipsoidal shape, the longer length R_1_ is determined on a cluster and then the shorter R_2_ is evaluated at the center point of the R_1_ at a perpendicular direction. Arithmetically averaged cluster sizes from 10 clusters are summarized using standard deviation (Fig. S1). Increased PEO to AuNP values from ×10, ×50 to ×100 (PEG/AuNP) can enhance the size of the clusters.

Figure 1b presents the small-angle X-ray scattering (SAXS) results conducted at the 4C beamline at the PAL with the designed nanocomposite networks in aqueous solution. Depending on the molecular weight and architecture, aqueous PEO solutions exhibit unique physical property: the solubility decrease and/or phase separation occurs above the critical temperature (lower critical solution temperature, LCST)^16^,^17^. At a fixed PEG concentration (100 mmol/L), temperature-responsiveness of the designed nanocomposites is compared at three different temperature conditions (20, 40 and 60°C). A characteristic *q* value (q* = 0.4 nm^−1^) indicates that the solubility of the nanocomposite network decreases according to the increase in temperature from 20 to 60°C, exhibiting the characteristic LCST. As temperature increases further, it moves to a lower *q* region, resulting in the formation of large structures. However, the q* point moves to a higher *q* region as the incorporated PEO amount increases (Figure S2) The organic−inorganic hybrid composites exhibit deviation from the typical elasticity of rubber, due to characteristic contribution of individual components. The designed nanocomposite networks are composed of dual domains, different from typical responsive materials of isotropic scale changes. The X-ray scattering intensity I(*q*) is experimentally determined as a function of the scattering vector *q* whose modulus is given by *q* = (4π/λ)sin(θ/2), where λ is the X-ray wavelength and θ is the scattering angle. Since our composites are microscopically isotropic the intensities will depend only on the modulus of *q* that for SAXS is given by *q* ≈ (2π/λ) θ. From the obtained peaks at the SAXS profile, the size-definable structures marked by *q*_1_ = 2.3 nm^−1^, *q*_2_ = 2.6 nm^−1^, and *q*_3_ = 3.0 nm^−1^ are generated at 60, 40 and 20°C, exhibiting larger size formation at increased temperature conditions.

Based on the obtained SAXS results, temperature-responsive nano-scale networks are suggested as illustrated in Figure 1c (left). The correlation length (ζ), the distance between the junction points of the network, changes according to temperature. Compared with the lower temperature condition of highly swollen state [I], the ζ values increase due to the shrinkage at an increased temperature [II]. The selected permeates pass the nanocomposites more effectively through larger ζ [II] with increasing temperature than through smaller ζ at a lower temperature [I]. The designed nanocomposites are embedded in poly(vinyl alcohol) (PVA) gel matrix (3wt%, M_w_~72,000 Merck), keeping the temperature-responsiveness of nanocomposites without leakage and shape deformation^18^. PVA matrix is ideal as a nanocomposite matrix, because no significant swelling or shrinking occurs under the designed experimental conditions. As the temperature increases several times above and below the transition point, the release of nanocomposites releasing from the PVA matrix is not detected. The designed nanocomposites in PVA matrix exhibits differentiated paths for the selected permeates according to the temperature condition^5^.

### Controlled mass transport through responsive nanocomposites

**Figure 2:**
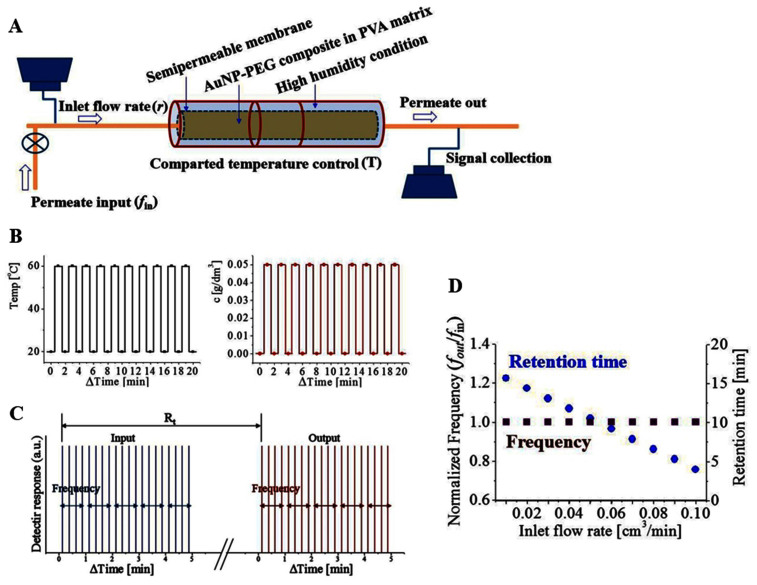
(a) Experimental set-up for mass-transport. Permeate molecules of periodic injection (*f*_in_) are loaded with a controlled flow rate (*r*). (b) Temperature control (left) and molecular signal detection (right) are highly synchronized, indicating stably optimized system for mass transport investigation. (c) Retention time (R_t_) during which the permeate molecules are stay in the cell before they are detected at the outlet. At a fixed *r*, the input frequency (*f*_in_) and output frequency (*f*_out_) are kept same. (d) R_t_ decreases linearly, whereas normalized frequency (*f*_out_/*f*_in_) maintains unity according to *r*.

Mass transport is effectively controlled using the designed apparatus (Fig. 2a). Selected permeate molecules^19^,^20^ pass through the nanocomposites loaded in a designed diffusion cell. The outlet of permeates is a function of permeate input frequency (*f*_in_), inlet flow rate (*r*), and geometry of nanocomposite controlled by temperature (T). The concentration-controlled permeate solutions are loaded at a designed time interval to generate periodic input. Approximately 0.01 μL of 0.1 g/mL rhodamine 6G aqueous solution is loaded for one shot, and the number of shots in a minute is varied by changing the *f*_in_ from 1 min^−1^ to 8 min^−1^. The signal from the eluted permeates is recorded in a continuous mode (see Methods). The nanocomposite is embedded in the PVA matrix for molding into a cylinder (diameter: 0.1 cm; length: 10 cm). This cylinder-shaped nanocomposite pellet is loaded into a humidity-controlled 0.1 cm-thick glass tube. The tube has heating coils, and it is connected to the inlet pressure and signal detecting devices. The durability and repeatability of the nanocomposite are verified up to 500 min of successive mechanical repetitions. Thus, all the experiments in the previous study are performed under this condition (Supporting Information).

The time for the permeate outlet is normalized to eliminate the condition-specific R_t_. Therefore, the time is expressed as Δt = t−t_0_ in the graph, where the zero point (t_0_) is the time at which injected permeates are first detected as an outlet. At the increased temperature condition of 60°C, the permeate is released in the detector (on position), and the permeate release is stopped at 20°C (off position). The temperature-controlled part in the tube is compartmented using individual heating coils to generate spatio-temporal modulation. Thus the structures can be regionally controlled by artificial gating functions. The input and output signals are highly synchronized as shown in Figure 2b. The left graph is the temperature signal, while the right graph exhibits detection signal of the outlet. The interval of the input signal is controlled to be longer than 15 s to provide sufficient time for the responsiveness of the nanocomposite. With carefully controlled experimental conditions, the results exhibit sharp synchronization responding to the applied temperature stimuli.

At the inlet flow rate (*r*) of 10 μL/min (10^−2^ cm^3^/min), proper repetition signals of rhodamine 6G are obtained for a periodic pattern beyond the characteristic retention time (R_t_) during which permeates are retained inside the diffusion cell (Figure 2c). By carefully controlling the volumetric inlet flow rate (*r*) for stable base line, modulated signals of the injected permeate molecules are obtained at a designed time interval. A sharp and discrete outlet signal is verified for a detectable concentration of permeate at a given experimental condition. As long as the flow rate (*r*) is fixed, normalized frequency of outlet to inlet (*f*_out_/*f*_in_) maintains unity (Figure 2c and 2d). However, the R_t_ decreases linearly as *r* increases (Figure 2d). R_t_ exhibits a typical linear relation according to the van Deemter equation as follows: *L* ~ *D*_1_ + *D*_2_/*r* + *D*_3 _*r*. In this equation, *L* is the permeate path length, *r* is the flow rate, *D*_1_ is the multiple diffusion parameter, *D*_2_ is the longitudinal diffusion and *D*_3_ is the mass transport parameters^43^. When the mass transfer effect is dominant, which corresponds to the system designed in this study, R_t_ is inversely proportional to the *r* in a linear relation. When the flow rate is controlled from 0.01 to 0.1 cm^3^/min, R_t_ changes from 16 min to 4 min (Figure 2d). When the total volume of the column as of 0.08 cm^3^, the efficiency factor (*H*) passing through the designed nanocomposite is close to 2 based on the following relation: *H* = *L* × [(R_t(permeate)_ – R_t(solvent)_)/R_t(solvent)_].

### Flow-controlled mass transport

**Figure 3:**
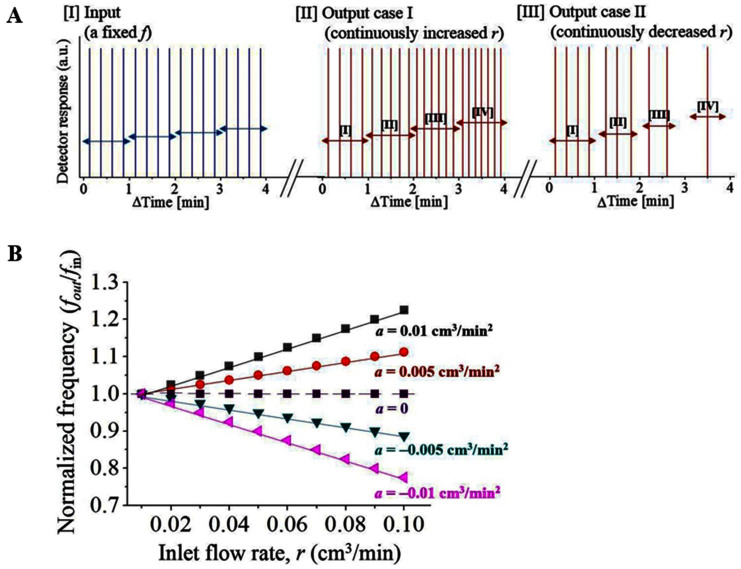
(a) Representative result of *f* change according to the designed *r* (*a*, acceleration). [I] Input at a fixed *f* and designed *r*. [II] Output case I: *f*_out_ increases with continuously increasing *r*. [III] Output case II: *f*_out_ decreases with continuously decreasing *r*. (b) Graphical summary of the results obtained in (a). Without acceleration (*a* = 0), *f*_out_/*f*_in_ maintains unity according to *r*. *f*_out_/*f*_in_ linearly increases with positive *a*, but linearly decreases with negative *a*. These results exhibit typical particle-like behavior of permeate molecules under acceleration caused by increased *r*. Considering the fixed path-length of the system, the result reflects the linear mass-energy relation.

Representative synchronized frequency patterns are displayed where the two inlet (*f*_in_) and outlet (*f*_out_) frequencies are highly synchronized (Figure 2B and 2c). At different *r* values (0.01 to 0.1 cm^3^/min), the normalized frequency (*f*_out_/*f*_in_) pattern is constant (*f*_out_/*f*_in_ = 1) as long as the acceleration rate is zero (*a* = d*r*_in_/dt = 0). However, when *r*_in_ is continuously changed at a fixed acceleration (*a* = d*r*_in_/dt), *f*_out_/*f*_in_ varies systematically (Figure 3a). Under the given condition, the variation of *f*_out_/*f*_in_ exhibits linear relation according to *a* (Figure 3b). At a given pass length condition (*L*), this linear relation with *a* directly indicates the linear proportionality to the kinetic energy. Therefore, the designed mass (rhodamine 6G) flow behaves as a conventional mass for which the general physical laws are applied: *E*(energy) ~ *F*(force) × *L*(length) ~ *m*(mass) × *a*(acceleration) × *L*. As *r*_in_ continuously increases or decreases at a constant permeate injection frequency (*f*_in_ = 4 min^−1^), *f*_out_ increases or decreases accordingly. The results for two representative cases are displayed in Figure 3a. As *r* changes with different *a* = ±0.005 cm^3^/min^2^ and *a* = ±0.01 cm^3^/min^2^, the variations in *f*_out_ are displayed at the middle and the right graphs in Figure 3a. Under no acceleration (*a* = 0), the inlet frequency (*f*_in_) and the first outlet frequency (*f*_out_) has no difference (*f*_out_/*f*_in_ = 1). The normalized frequency (*f*_out_/*f*_in_) exhibit linear proportionality for continuous change of *a*. This finding is attributed to to the mass-energy relation where the energy (*E*) is proportional to volumetric acceleration (*a*): *E* ~ *a*. Therefore, the volumetric acceleration/deceleration, rather than the absolute flow rate, has a significant influence on the permeate output. Even for densely packed viscous soft condensed matter media designed in this study, the energy (E) relation is satisfied as the permeates freely pass through
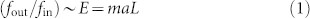


The passing molecules are accelerated following the conventional mass-energy relation applied in typical particle-like physical behaviors.

### Mass transport by contractile actions of one-valve system

**Figure 4:**
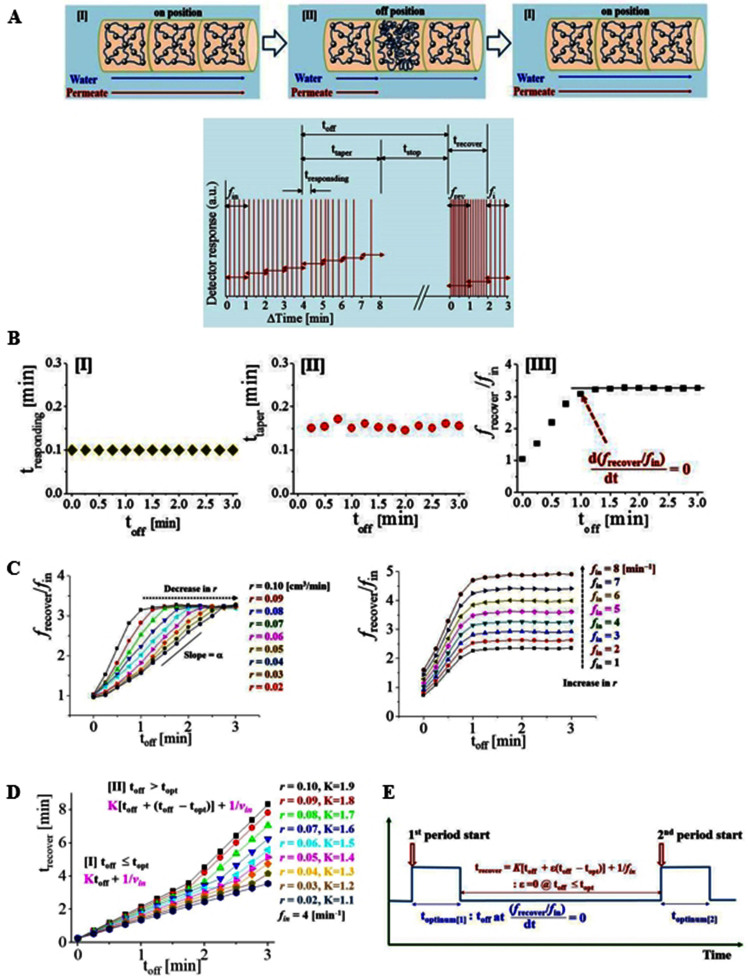
(a) Design of the contractile action on flow. By repeating on−off−on position, one valve function is designed (upper). Representative data of the one-valve system. During the designed off-time (t_off_), the inlet flow stops. During the taper time (t_taper_), detection of permeate molecules slows followed by complete stop-time (t_stop_). Right after the t_off_ starts, there is a responding time (t_responding_) at which the flow stops suddenly and generates back pressure inside the cell. According to the inlet frequency (*f*_in_), the outlet frequency is modulated. Right after the t_off_, the first maximum outlet frequency is determined as recover frequency (*f*_recover_) after that i^th^ frequency is determined in a sequence. (b) [I] t_off_ vs. t_responding_ [II] t_off_ vs. t_taper_. t_responding_ and t_taper_ are independent of t_off_, reflecting system-specific features. [III] t_off_ vs. maximum normalized frequency (*f*_recover_/*f*_in_). The frequency increase is saturated at a critical point, so the optimum time (t_optinum_) is determined. (c) t_off_ vs. *f*_recover_/*f*_in_ with changing *r* (left) and *f*_in_ (right). The critical point move to higher t_off_ by decreasing *r*, whereas a higher *f*_in_ contributes to the increase in the magnitude of *f*_recover_/*f*_in_. (d) Fitting results of the t_recover_ vs. t_off_ in the two regions: [I] in the range of t_off_ ≤ t_opt_, K(*r*) t_off_ + 1/*f_in_* relation is satisfied. [II] in the range of t_off_ > t_opt_, K(*r*) [t_off_ + (t_off_ − t_opt_)] + 1/*f_in_* relation is applied. The proportional factor K is also a function of *r*. (**e**) Summary of one periodicity composed of t_off_ and t_recover_ as a function of t_off_, t_optinum_, *f*_recover_ and *f*_in_.

With a fixed *f*_in_ = 4 (min^−1^) and *r* = 0.01 cm^3^/min, temperature at the middle of the designed nanocomposite pellet is controlled (Figure 4a). The off position in the valve function is provided by switching the temperature from 60°C to 20°C. The cooling process is only applied to the middle section on 2 cm length region (out of 10 cm total tube length) to induce temperature gradient. The frequency maintains a regular pattern (4 min^−1^) before changing to the off-state. However, during the responding time (t_respond_), permeate movement is suddenly delayed by the contractile actions in the off-state. This phenomenon is caused by the swelling procedure of the nanocomposite, which increases the back pressure caused by sudden water absorption. After the abrupt stop of permeate movement, the decreased water flow induces an incremental decrease in *f*_out_, forming a tapering zone (t_taper_). Once most of the permeates exit the off-zone, the permeate elution is completely stopped until the designed t_off_ ends. When the on-position state and flow are both regained after t_off_, the permeates are detected in a high frequency (*f*_recover_) because of accumulative congestion during the period of t_off_. The frequency stabilizes during the recovering time (t_recover_) to regain original frequency (*f*_in_ = *fi*), where fi is the *i*th frequency after *f*_recover_.

**Figure 5:**
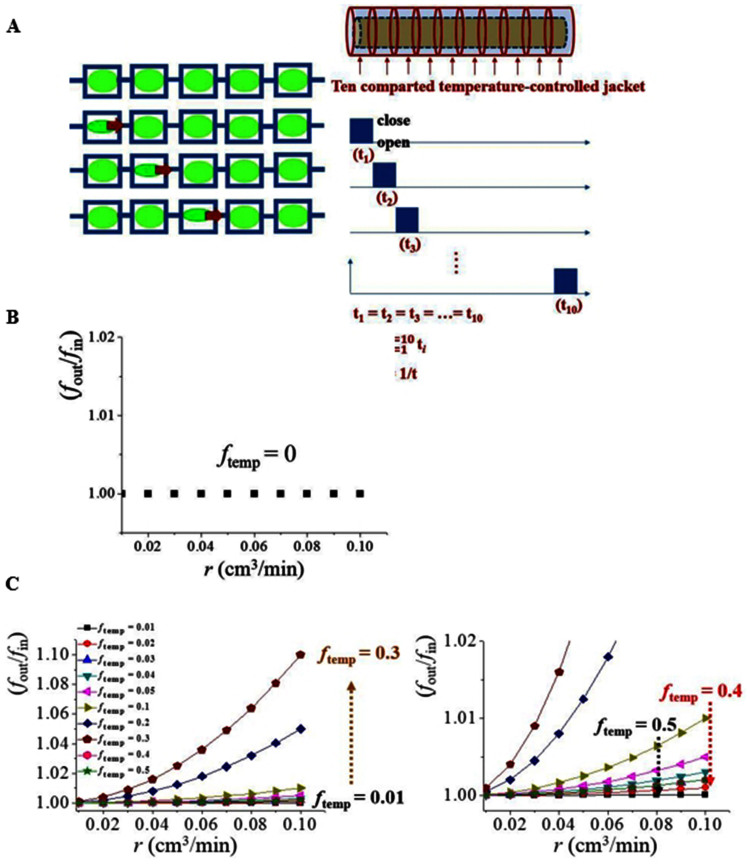
(a) Schematic illustration of the multi-valve system of sequential contractile actions. The cell is divided into 10 units of individual temperature control. The time for each unit is designed to be same as t_1_ = t_2 _ = t_3_……. = t_10_. The temperature frequency (*f*_temp_) is determined as inverse proportionality of the total 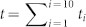 time taking for one cycle. (b) Without *f*_temp_ introduction, the system exhibits stable (*f*_out_/*f*_in_) = 1. (c) With increased *f*_temp_ from 0 to 0.3 (min^−1^), the *f*_out_/*f*_in_ increases proportionally to *r*^2^. However, *f*_out_/*f*_in_ decreases significantly from *f*_temp_ = 0.4 min^−1^. Therefore, an optimum *f*_temp_ is necessary to maximize the *f*_out_/*f*_in_.

With varying duration of t_off_, the resulting *f*_out_ of the designed systems is observed accordingly (Figure 4b). t_off_ is controlled from 15 s to 3 minutes in the middle of the stable flow at a fixed *r*. t_respond_ [I] and t_taper_ [II] are independent of t_off_, which reflects the characteristic property of the nanocomposite itself. Based on the increase in the t_off_, the first maximum recovering frequency (*f*_recover_) right after the t_off_ is normalized by the inlet frequency (*f*_recover_/*f*_in_) [III]. This normalized value is saturated from a specific point (d(*f*_recover_/*f*_in_)/dt = 0) at which the optimum off-time (t_opt_) is determined. This finding implies a limit to increase the mass transport rate continuously by increasing the t_off_. Thus an optimized contractile action exist. To find the critical point from which the increase of the mass transport is limited (or saturated), variations in *f*_recover_/*f*_in_ is investigated with varying parameters (Figure 5c). With decreasing the *r*, the time to reach the critical point takes longer. Before reaching the critical point, the *f*_recover_/*f*_in_ relation follows an approximate liner relation with a definable slope α as a function of *r*: (*f*_recover_/*f*_in_) = α(*r*) t_off_ + ϕ, where ϕ is an intercept (Table S1). However, high *f*_in_ only increases the magnitude of the *f*_recover_/*f*_in_ whereas the critical point remains unchanged. Therefore, to determine the optimized t_off_, *r*_in_ is exclusively decisive, and *f*_in_ contributes to the magnitude of the *f*_recover_/*f*_in_. Therefore, the mass transport is controlled by the energy impartment conveyed by the flow acceleration. Since *f*_recover_/*f*_in_ is linearly proportional to *r*, the following relation is reasonably satisfied:
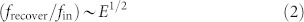


Compared with Eq. (1), which is applicable to stable flows linearly proportional to *E* without contractile action, the normalized maximum outlet frequency (*f*_recover_/*f*_in_) is proportional to the half power of *E* when a contractile action is applied.

Figure 4d shows the variations of the time duration elapsed to recover the original frequency (t_recover_) plotted by t_off_. The time duration is classified into two regions, namely t_off_ and t_opt_:
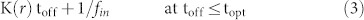

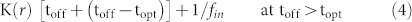


In Eq. (3), t_recover_ is linearly proportional to t_off_. In Eq. (4), the difference between the actually performed off-time and the optimum off-time (t_off_ − t_opt_) is additionally inlcuded. t_optinum_ is the suggested time for which the mass transport rate is maximized, and an extended time period does not increase the mass transport rate. Therefore, t_recover_ is necessary to deliver the overloaded masses. The obtained experimental results are fitted by the linear relation of Eq. (3). In additin, the proprotional factor K(*r*) is a fucntion of ***r*** satisfying a liner relation with *r*. An intercept value of 1/*f*_in_ explains that the t_recover_ is inversely proportional to the *f*_in_ in all the cases in this study. The concept of the optimized periodicity thus summarized in Figure 4e. One period is composed of t_optinum_ and t_recover_ which are the functions of the t_off_, *f*_recover_ and *f*_in_.

### Mass transport by contractile actions in a series of multi-valve system

Mass transport is controlled by consecutive repeated contractile actions (Figure 5a). With a series of the 10 compartments, each section is sequentially heated to generate a partial blockage during the interval time (t_interval_) from t_1_ to t_10_ under the conditions of t_1_ = t_2_ = t_3_ = … = t_10_. When the contractile actions are performed in a series, a pushing effect on a specific point occurs, illustrated as arrows on the left-side. Heating is applied on each compartmental section of the temperature-controlled tube on the right in a series, so consecutively moving contractile actions can be generated. The stability of the system is verified by checking whether the normalized frequency (*f*_out_/*f*_in_) of the system maintains unity without temperature modulation (Figure 5b). When the total elapsed time t_interval_ = Σt*_i_* (*i* = 1, 2, 3, …10) is determined, then the temperature frequency is defined as *f*_temp_ = 1/t_interval_. When a partial contractile action is applied in a series (Figure 5c), the mass transport can be either promoted or prohibited. With the increase in inlet flow rate (*r*), *f*_out_/*f*_in_ value increases until a specific frequency (i.e., *f*_temp_ = 0.3) and then significantly drops down. In addition, *f*_out_/*f*_in_ is proportional to the square of the *r* as curve-fitted in the graph. The experimental conditions and the fitting results are available in Supporting Information (Table S2). This result indicates that mass transport is accelerated linearly proportional to the energy as follows:
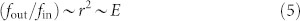


Therefore, high synchronization of the contractile actions performed in a series is necessary to match with the fluid flow rate.

## Discussion

Mass transport has been investigated typically based on diffusion process for which Fick's law is applied^21^. Transport through small-scale pores, such as reverse osmosis membranes, has been described by the solution–diffusion model^22^ and other modelling techniques^23^. In addition, small ions and large-scale colloidal particles in size ranges of 1 to 100 nm have been analyzed in a single theoretical framework^24^. Although the discrete nature of individual nanoscale molecules is evident, the main transport phenomena in nanofluidic systems are explained based on the continuum and mean-field approaches. Unlike these diffusion-dominant passive concepts, a new molecular transport is recapitulated in this study based on the acceleration by repeated contractile action in fluid flow. Depending on the contractile styles (one-valve and continuous multi-valve systems), the amount of energy imparted by the contractile actions is different. Under continuously increased flow rate (with a fixed acceleration) or sequential contractile multi-valve motion, the mass transport efficiency expressed by normalized frequency (*f*_out_/*f*_in_) is linearly proportional to the energy: (*f*_out_/*f*_in_) ~ *E*. Regarding the contractile action using a one-valve system, the relation (*f*_out_/*f*_in_) ~ *E*^1/2^ is satisfied.

Contractile energy is employed in many natural and artificial systems. Blood flow activated by heart beats and undulatory actions of intestines are representative contractile-promoted actions occurring in biological systems. This repeated contractile phenomena actually occur in the human body. The natural defensive mechanism of the human body such as response to the hypotension hemorrhage and blood vessel occlusion, might be explained based on the physical phenomena occurring in the optimal model. In practical artificial systems, intermittent flow controlled systems are generally relevant to this concept. We can successfully reenact the important dynamic behaviors occurring natural systems by effectively employing stimuli-responsive soft condensed materials^25^. This study would contribute to the basic and general understanding on the optimal mass transport in the dynamics-promoted flow systems.

## Methods

### Permeate molecule transport through AuNP-PEG nanocomposites

The volume and height of the AuNP-PEG nanocomposite embedded in the PVA matrix (3 wt%, Mw = 72,000; Merck) is designed as a pellet (diameter = 0.1 cm; length = 10 cm). The nanocomposite pellets are loaded in a thin-walled (0.01 mm) glass tube (Hampton Research, CA, USA) in which the humidity and temperature are carefully controlled. Water flow rate (*r*) and mass transport frequency (*f*) are controlled through the designed nanocomposites. Rhodamine 6G is an ionic molecule sensitive to laser-based detection. It exhibits a dark orange color in water, so it is used as an effective tracer in water flow. With high water solubility (400 g/L), rhodamine 6G is directly dissolved in water and used as a saturated solution. Concentration-controlled permeate solutions are injected into the inlet line at the designed time interval (Micro-Liter OEM Syringe Pump Modules, Harvard Apparatus, MA, USA). A series of eluted permeates are measured and recorded using a spectrophotometer (2489 UV/visible detector, Waters, MA, USA). Empower 3 Chromatography Data Software is employed for data analysis. Spectra are obtained at a scan rate of 100 nm/s, and intensities are collected at an interval of 0.5 nm. For long-term and quick scanning, the wavelength range is narrowly controlled for rhodamine 6G spectra at an excitation wavelength of 485 nm. Scanning is performed every 5 s so that the possible maximum frequency designed in this study is evaluated as 12 min^−1^. All the spectra are obtained at 20°C. To precisely control the local temperature of the designed nanocomposites, a multi-point temperature controller (MPC, Briskheat Corp., OH, USA) with resistance heating coils made of coiled chromel wire (MOR Electric Heating Assoc. Inc., MI, USA) is employed.

### Small-angle X-ray scattering (SAXS)

SAXS. Synchrotron SAXS measurements are obtained at the 4C beamline at PAL equipped with a position-sensitive two-dimensional (2D) detector. Two energy settings are employed for wavelength modulation: 10 (0.0675 nm^−1^) and 18 keV (0.1217 nm^−1^). Samples of 1 mm-thick are used by stacking five 200 μm-thick Si wafer with SiN3 sample window (window thickness is 1 μm so that it hardly contributes to the sample thickness). The sample-to-detector distance (SDD) of 4 m covers the q range of 0.0679 nm^−1^ < q < 1.64094 nm^−1^, where *q* = (4π/λ)sin(θ/2) is the magnitude of the scattering vector and θ is the scattering angle. The *q* range is calibrated using polystyrene-*block*-poly(ethylene-*ran*-butylene)-*block*-polystyrene (SEBS) (*q* = 0.19165 nm^−1^). On the other hand, the 1 m SDD covers the *q* range of 0.346 nm^−1^ < *q* < 7.68039 nm^−1^. The *q* range is calibrated using silver behenate (*q* = 1.052 nm^−1^). During the measurements, the temperature is precisely controlled at an isothermal condition of 20°C if no heating plan is involved. To increase the sample temperature to 40°C and 60°C, programmed heating coil is employed to connect to the copper-based sample holder body. A W/B4C double multilayer monochromator is installed to deliver monochromatic X-rays with a wavelength of 6.75 nm (18360 keV) and spread of Δλ/λ = 0.01. The 2D scattered X-rays are recorded by a CCD camera (Mar CCD, Mar USA, Inc., CCD165). The collected SAXS data are corrected by subtracting the background and empty cell scattering.

### Synchrotron X-ray nanoscopy (XN)^26^

Experiments are carried out at the 7C beamline of PAL. The X-ray source of 10^11^ photons/μm^2^/sec consists of undulator with 20 mm period and 70 poles. The beam size is about 100 μm × 100 μm at 7 keV. The X-ray source is radiated from a 3 GeV bending magnet and then monochromatized using a Ge(111) DCM. To achieve focused images, monochromatic X-ray beam of nominally selected 7 keV is focused on the sample using a condenser zone-plate (CZP, 1 mm dia. Beryllium refractive compound lenses) with innermost and outermost diameters of 4 nm and 100 nm, respectively. The primary X-ray image is magnified 50 times with an objective zone plate lens (140 μm innermost and 50 nm outermost diameter, W). It is then converted into a visible image on a thin scintillator crystal (Tb:LSO, 20 μm thickness). The visible image is further magnified ×20, using an optical microscope. This provides a total ×1000 magnification of image on a cooled CCD camera (Princeton Instrument VersArray 1300B cooled CCD) of 1340 × 1300 pixels, which corresponds to an equivalent FOV of 21 × 21 μm^2^.

### Synchrotron X-ray microscopy (XM)

Synchrotron X-ray images are captured at 6D beamline of PAL. The X-ray source is a bending magnet with critical energy of 8.7 keV at 3GeV electron energy operation. The white beam is attenuated by polished beryllium (Be) of 0.5 mm thickness and polished Si wafer of 1 mm thickness. The primary X-ray image is converted into a visible image on the thin scintillator crystal CdWO_4_ of 100 μm thickness. X-ray images are captured using a CCD camera (Vieworks, VH-2MC). With a 10× objective lens attached in front of the camera generates the field-of-view of 1.2 mm × 0.9 mm in physical dimension. The size of each pixel is about 0.74 μm.

## Author Contributions

S.A. and S.J.L. developed the concepts. S.A. designed and perform the experiments. S.A. analyzed the results and wrote the paper. All the authors confirm the final version of the manuscript.

## Supplementary Material

Supplementary Information Supporting information and data
