# Supplementary material for: Optimum periodicity of repeated contractile actions applied in mass transport
Source: Sci Rep. 2015 Jan 27;5:7800. doi: 10.1038/srep07800 (PMC4306965; doi:10.1038/srep07800)
Supplement: Supplementary Information — Supporting information and data [file srep07800-s1.doc]

**Optimum periodicity of repeated contractile actions applied in mass transport**

Sungsook Ahn^1,2^ and Sang Joon Lee^1,2,*^

^1^Biofluid and Biomimic Research Center, ^2^Department of Mechanical Engineering

Pohang University of Science and Technology, Pohang, 790-784, Korea.

*Correspondence

Tel: +82-54-279-2169, Fax: +82-54-279- 3199, E-mail: sjlee@postech.ac.kr

**Keywords**

Periodicity, Contractile flows, Mass transport, Stimuli responsive, Nanocomposite

**Supporting Information**

**Preparation of spherical AuNPs of 20 nm in average diameter.** To prepare gold nanoparticles (AuNPs), gold chloride (III) trihydrate (HAuCl_4_⋅3H_2_O) is dissolved in DI Milli-Q water (1.0 × 10^-3^ mol/L) under refluxing. Sodium citrate tribasic dihydrate solution in DI water (4 × 10^-2^ mol/L) is added to the above solution. Reaction completion is detected by color change from yellow to wine red. Boiling condition is further maintained for 15 min after the color change is completed and then cooled to room temperature. The AuNP solution is dialyzed overnight using Spectra/Por®7 membrane (1,000 Da cut) against DI Milli-Q water to remove excess sodium citrate tribasic dihydrate. The AuNP solution is placed on a copper grid and then dried under air at room temperature for transmission electron microscopy (TEM) (JEOL Cs-corrected HR-TEM, JEM-2200FS). Given that the organic layers of the AuNPs are not clearly detected by TEM, gold cores with high electron density in AuNPs are captured distinctively. From this TEM image, the average diameter of the formed AuNPs is measured to be approximately 20 nm.

**Preparation of interconnected AuNPs by PEGs.** Thiol end-capped polyethylene oxide (PEO) ligands are added at the second step. Bi-functional and four-armed PEGs are purchased (Laysan Bio, Arab, AL, USA) and utilized without further purification. The aqueous surface-modified AuNP stock solutions are adjusted to a concentration of 2.4 × 10^18^ AuNPs/m^3^ in consideration of the diameter of AuNPs (average diameter of 20 nm). After measuring the size of the AuNPs, a designed PEO solution is added to the above aqueous solution to interconnect the citrate-covered AuNP and stirred at room temperature or higher (between 50 °C and 60 °C) for 6 h to 12 h. The un-reacted residue ligands are minimized to less than 1 ppm by checking the aliquot of the samples. The AuNP solutions are dialyzed overnight by Spectra/Por®7 membrane (25 KDa cut) against Milli-Q water for purification. For the standard concentration of the AuNP stock solution of 1.0 mmol/L, the concentration of the ligand stock solution is varied to 10, 50, and 100 mmol (Figure S2).

The physical properties of the AuNP clusters formed by PEG interlinking are investigated by UV-vis spectral analysis. Physically hybrid AuNP clusters typically red-shift (such as natural aging). The UV-vis spectra of the designed AuNPs tethered by a constant PEG chain length of 10,000, but in different linear, binary, and quaternary structures, are compared (Figure S1a). The AuNPs linked by linear PEG generate surface plasmon at approximately λ = 540 nm, which is similar to that of a single AuNP with diameter of 20 nm. However, the AuNPs linked by binary and quaternary PEGs shift to 610 and 630 nm, respectively. The UV-vis spectrum of the designed PEG-AuNP nanocomposite clusters closely resembles that of physically aggregated NPs. In addition, the 4PEG-linked clusters red-shift more effectively compared with the 2PEG-linked ones. The characteristic light-responsiveness of AuNP clusters varies according to the chemical structure of the interlinking molecules.

NPs are usually considered to perform a random walk on the lattice model. In addition, effective mobility of particles on the lattice may be controlled by specific particle-to-solvent step ratios. The lattice model illustrated in Figure S1b is adopted to describe the interlinked AuNP–PEG nanocomposite clusters. Each lattice contains one AuNP, and these lattices are diversely interconnected depending on the molecular weight and chemical structure of PEGs. Multiple PEG linkages are attached on the surface of AuNPs to make assemblies. The mono-tethered AuNPs are analogous to single-tailed surfactants or diblock copolymers, whereas the multi-tethered AuNPs work as junction points in the polymer networks. They also possess additional levels of complexity and anisotropy that are exploited in self-assembly. The crosslink density (ρ) of the fully-interlinked network is inversely proportional to the molecular weight between junction points (M_p_).

**Figure S1.** **(a)** UV-vis spectroscopy of the AuNPs of 20 nm in diameter linked by functional PEGs of different structures but with the same arm length of Mp = 10,000. The AuNPs linked by linear PEG 10,000 exhibit UV-vis absorbance at 540 nm, whereas those linked by binary 2PEG 10,000 and quaternary 4PEG 10,000 have far longer wavelengths of 610 and 630 nm, respectively. **(b)** A lattice model used to describe the structures of the AuNP-PEG nanocomposite networks. Each AuNP is located in a lattice, and PEGs interconnect with those AuNPs. (c) Variation in the cluster size of the AuNPs linked by binary functional PEG of Mp = 10,000 (2PEG 10,000) with concentrations of ×10, ×50, and ×100 (number of PEGs/number of AuNPs). The cluster size is averaged from 100 clusters in XNI images. Each number on the graph indicates the PEG concentration for each case. The standard deviation is marked as pink bars.

**(a) (b) (c)**

**
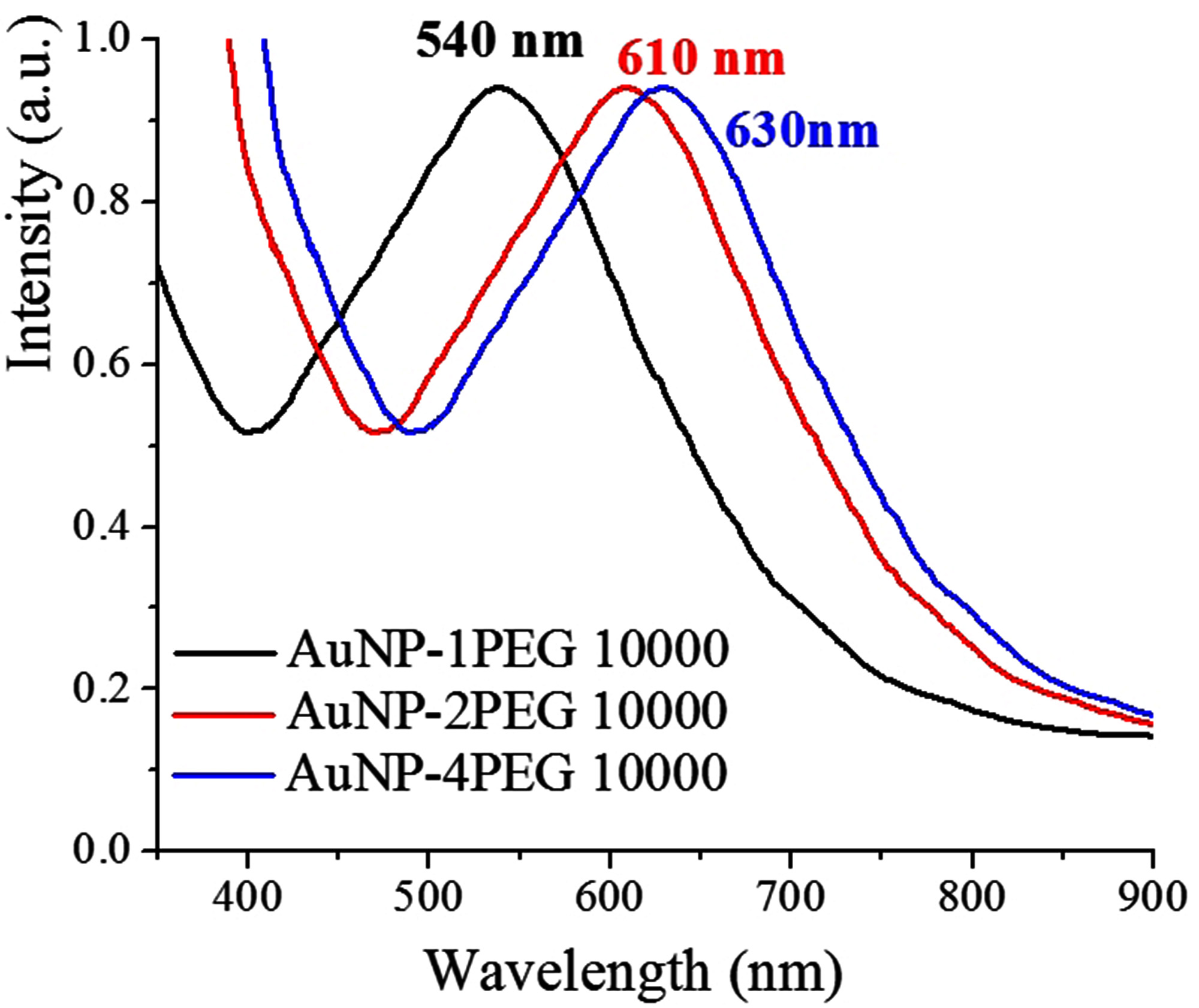

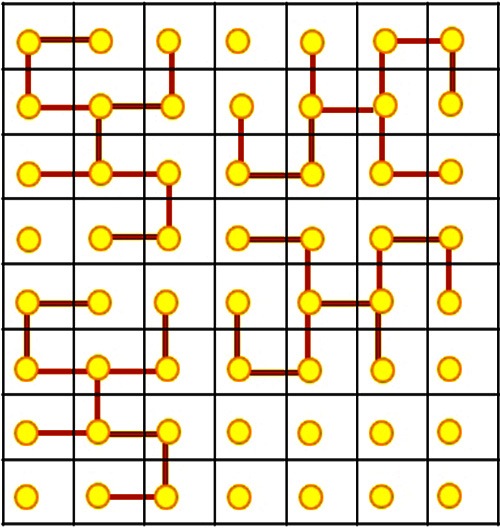

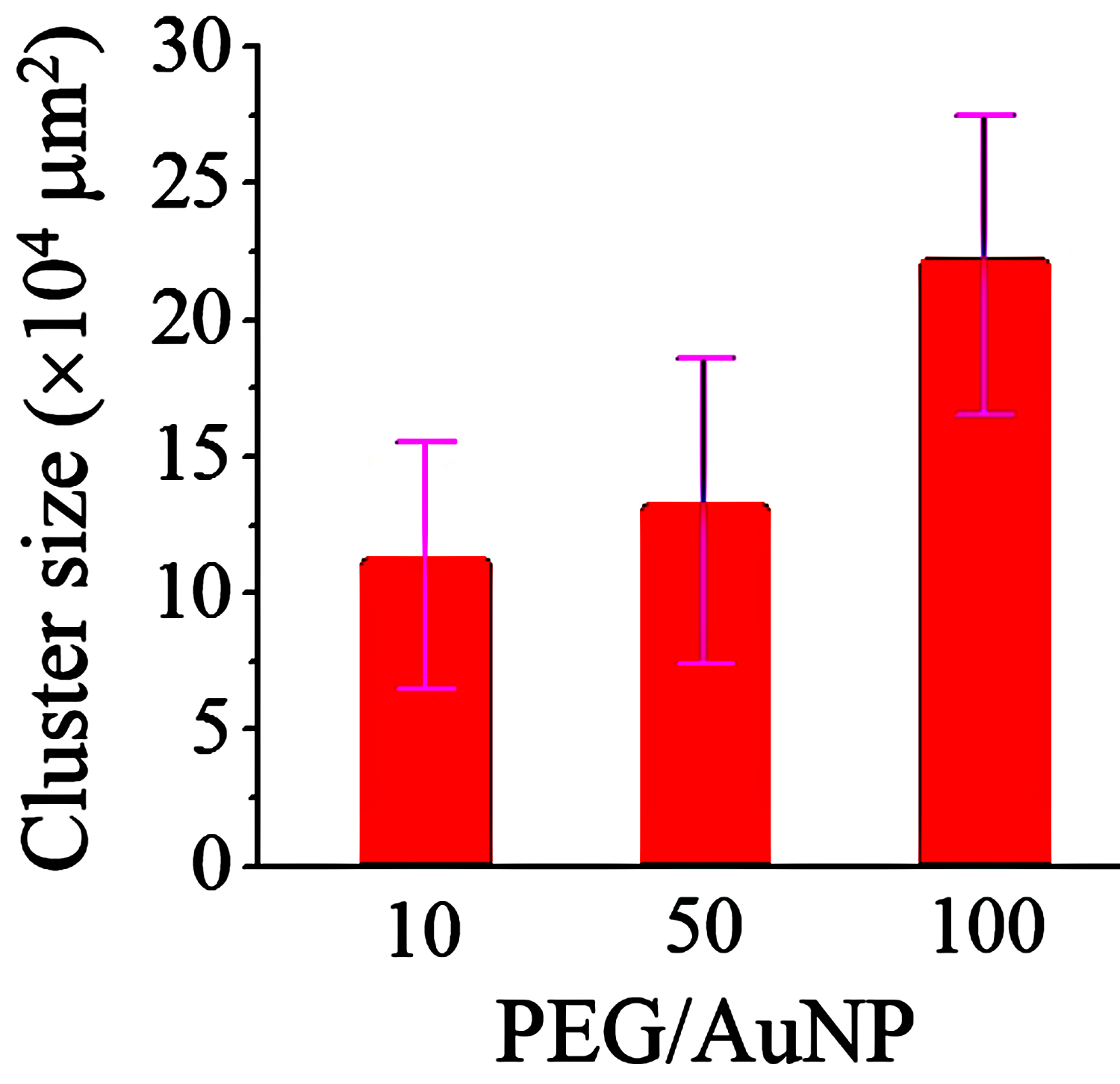
**

**Figure S2.** Effect of AuNP and PEG ratio on the structures obtained by SAXS. The aqueous surface-modified AuNP stock solutions are adjusted to a concentration of 2.4×10^18^ AuNPs/m^3^. Given that the standard concentration of the AuNP stock solution is 1.0 mmol/L, the concentration of the PEG ligand stock solution is controlled to 10 (×10), 50 (×50), and 100 mmol (×100). Beyond the critical *q** value, the systems are responsive to temperaturesranging from 20 °C to 60 °C. However, all the systems are stable below this *q** value. The *q** values shift toward a higher q region with increasing PEG amount for all systems. This finding indicates that the system responds to external stimuli from a small-sized region.

**
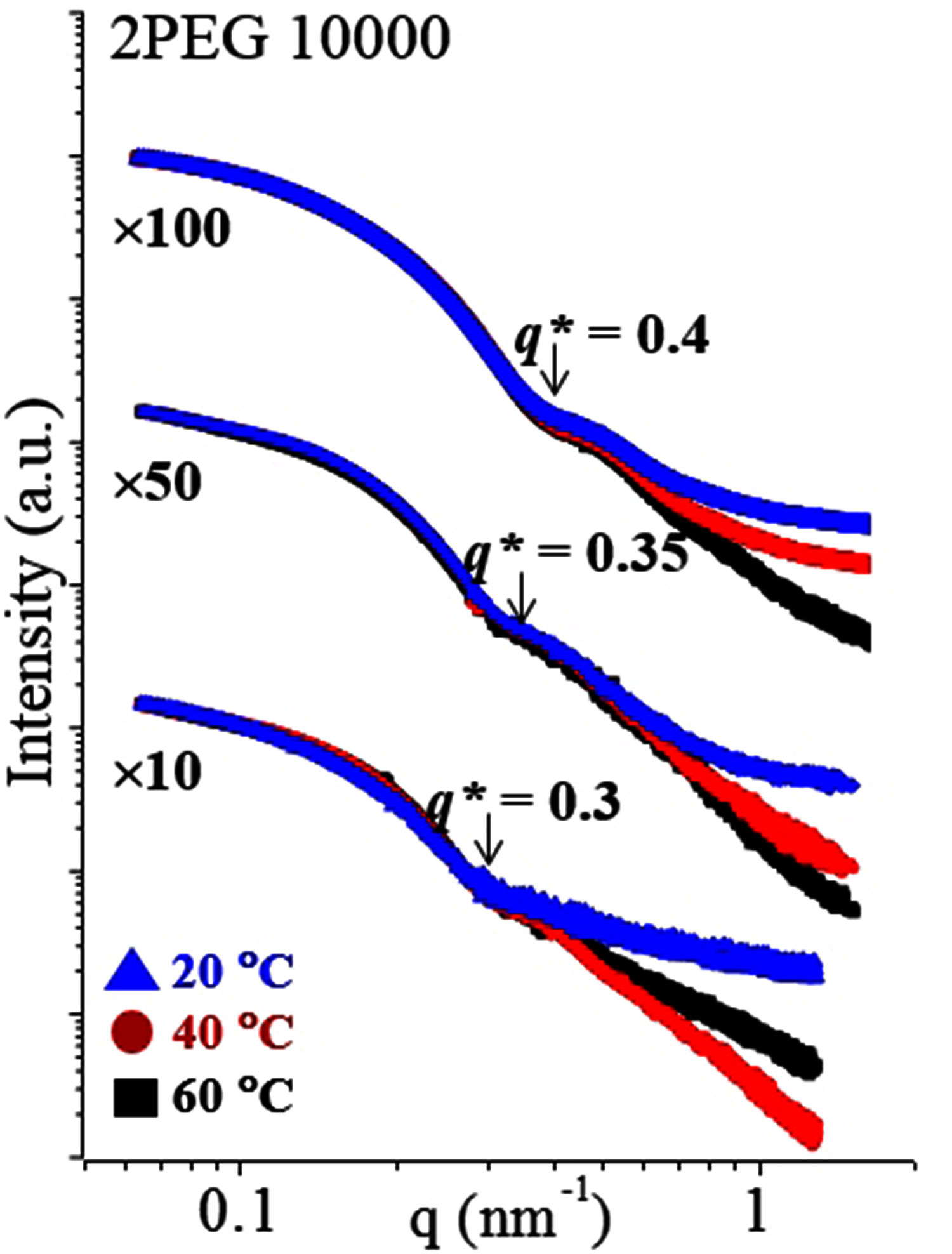
**

**Figure S3.** Variation in durability and repeatability of the designed nanocomposites. The temperature-triggered response of the designed nanocomposites and normalized elution time of permeates are coordinated up to 500 min of successive mechanical swelling–shrinking procedure. The two signals are highly synchronized


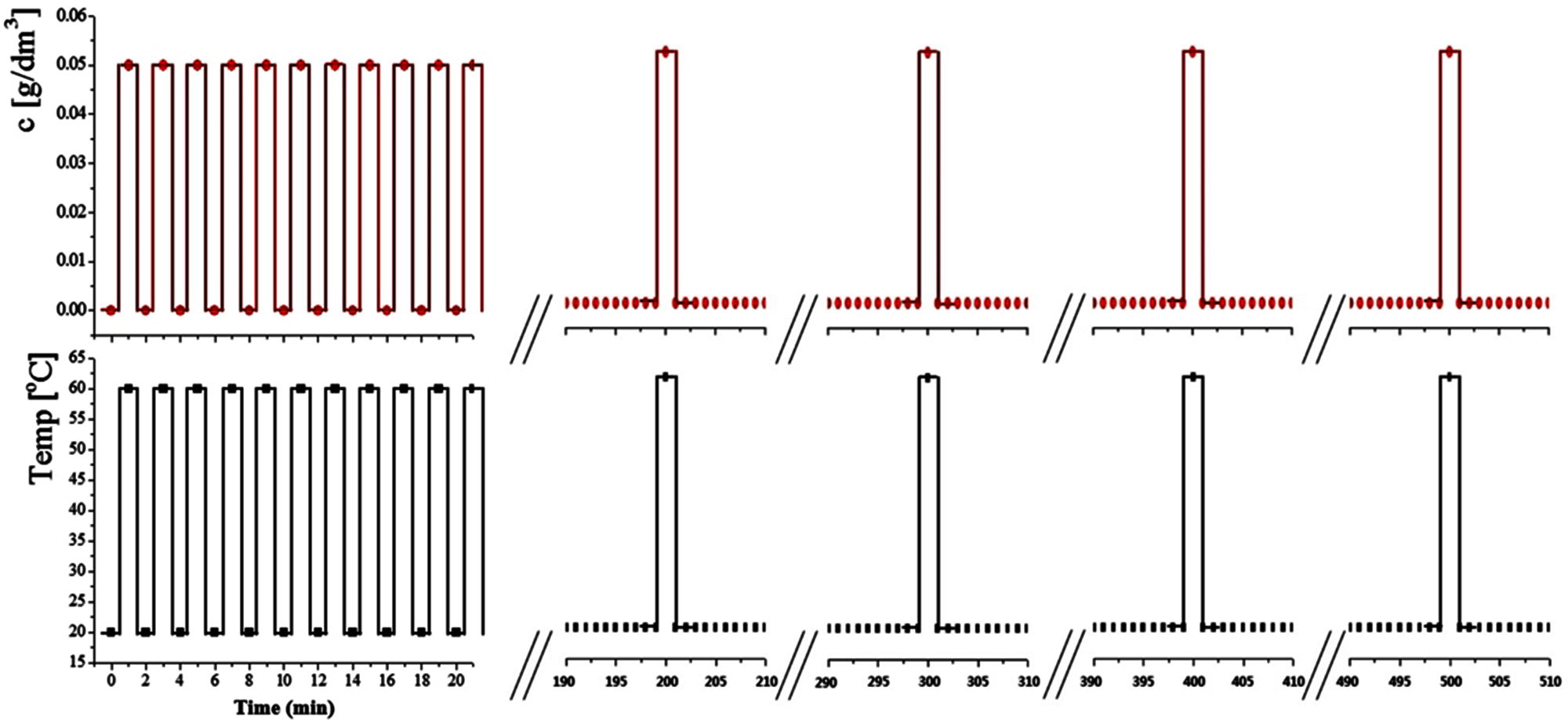


**Small-angle X-ray scattering (SAXS).** Synchrotron SAXS analysis is performed at the 4C beamline of PAL equipped with a position-sensitive two-dimensional (2D) detector. Two energy levels are employed for wavelength modulation: 10 keV (0.0675 nm^−1^) and 18 KeV (0.1217 nm^−1^). A samples of 1 mm thick is prepared by stacking five 200 μm-thick Si wafers with SiN_3_ sample window. The sample-to-detector distance (SDD) of 4 m covers the *q* range of 0.0679 nm^−1^ < *q* < 1.64094 nm^−1^, where *q =* (4π/λ)sin(θ/2) is the magnitude of the scattering vector and θ is the scattering angle. The *q* range is calibrated using polystyrene-*block*-poly(ethylene-*ran*-butylene)-*block*-polystyrene (SEBS) (*q* = 0.19165 nm^−1^). On the other hand, the SDD of 1 m covers the *q* range of 0.346 nm^−1^ < *q* < 7.68039 nm^−1^. The *q* ranges are calibrated using silver behenate (*q* = 1.052 nm^−1^). A W/B4C double multilayer monochromator are installed to deliver monochromatic X-ray beam with the wavelength of 6.75 nm (18360 keV) and spread of Δλ/λ = 0.01. The scattered X-rays are recorded by a CCD camera (Mar CCD, Mar USA, Inc., CCD165). The collected SAXS data are corrected by subtracting the background and empty cell scattering.


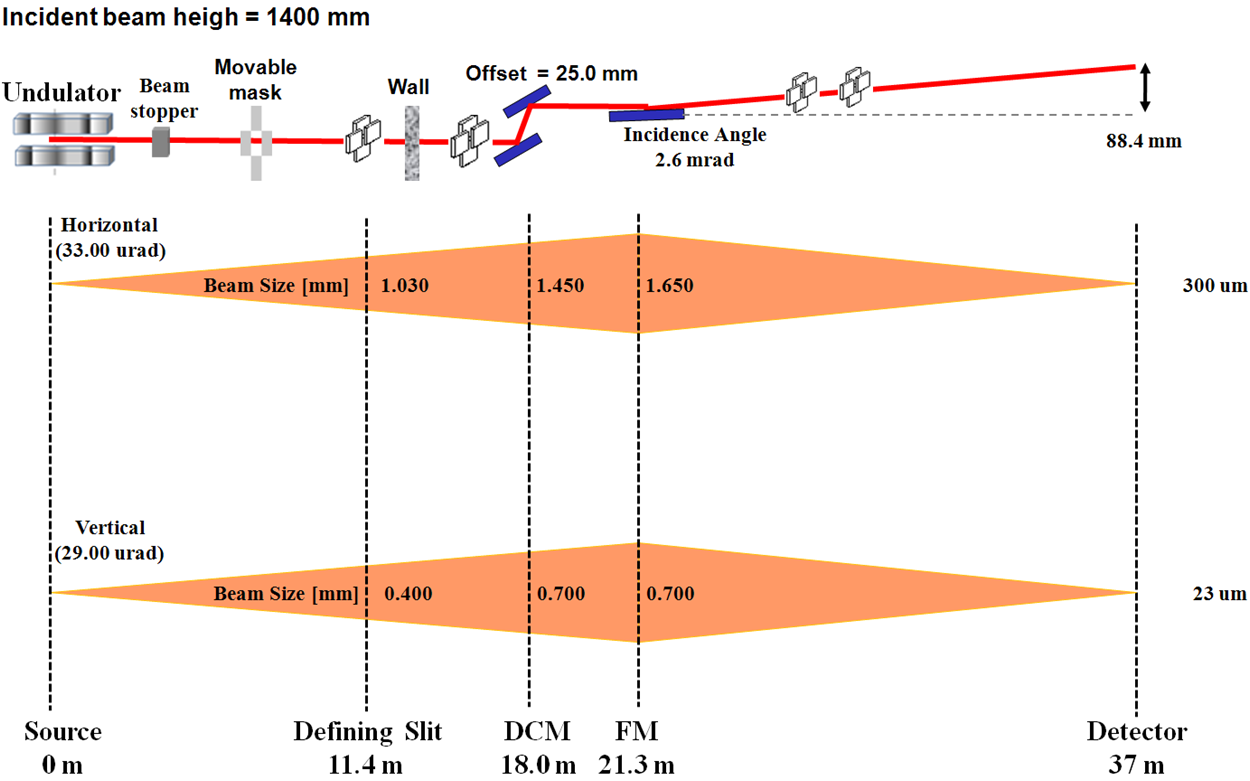


**Figure S4.** Experimental set-up for small angle X-ray scattering (SAXS) at PAL

**Synchrotron X-ray nanoscopy (XN)***.* Experiments are carried out at the 7C beam line of PAL. Fig. S5 shows the optical layout of the 7C XNI beamline along with a photograph of the main components. High-ﬂux X-rays are provided by means of a 1.4 m-long hybrid-type in-vacuum undulator with a period of 20 mm. In order to focus, or rather collimate, the divergent X-rays, ten beryllium parabolic CRLs of diameter 1 mm and effective aperture 0.6 mm (reduced by absorption), positioned 25.7 m downstream from the source, are employed. In order to reduce the spatial coherency and homogenize the illumination, a diffuser (rotating paper) is inserted in front of the sample. The sample is mounted on a three-axis piezo-driven scanning stage on top of an air-bearing rotation stage. The objective zone plate, of 50 nm outermost zone width, 140 μm diameter and 1.0 μm thickness, is made of tungsten. For Zernike phase contrast, a holed aluminium-ﬁlm phase plate of 3.78 μm is positioned near the back focal plane of the zone plate. The thickness is selected so as to phase shift the diffracted beam by π/2 and, thereby, make the sample image darker in the bright ﬁeld. The hole, of 10 μm diameter, is drilled by focused ion beams. The detector comprises a thin (18 μm) Tb:LSO scintillator crystal of 10 mm diameter and an X20 homemade optical microscope. The microscope is composed of an X20 objective lens and a CCD. The CCD has 4096 × 4096 pixels of 9 μm size. All of the optics, including the diffuser, pinhole, sample, zone plate, phase plate and detector, are installed on a 5 m-long 30 cm-thick granite plate to minimize external vibration.


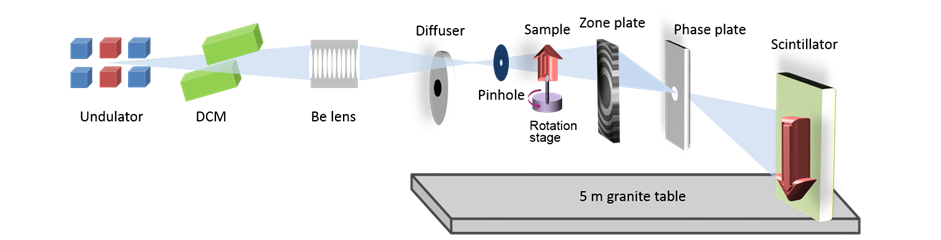


**Figure S5.** Experimental set-up for X-ray nanoscopy (XN) at PAL

**X-ray microscopy (XM).** Synchrotron X-ray images are captured at the 6D beamline of the PAL. The X-ray source is a bending magnet of 8.7 keV at 3GeV electron energy operation. The white beam is attenuated by a polished beryllium (Be) of 0.5 mm thickness and polished Si wafer of 1 mm thickness. The photon energy (E) is about 22 keV with energy resolution (ΔE/E) of 15.8 keV to 34.4 keV (~ 84 %). The brightness is ~2 × 10^12^ (ph/s/mm^2^) and the beam size is about 30 mm (H) × 5 mm (V). The sample is placed at 31 m downstream of the source approximately, whereas the detector is placed at 30 cm downstream of the objects. The size of X-ray beam illuminating the test sample is adjusted to that of the field-of-view by using a slit module to avoid unnecessary exposure of X-ray beam on the sample. An attenuator made of polished silicon (Si) wafers is located at the beam inlet of the experiment hutch on the pathway of X-ray propagation to attenuate light intensity, even when no image is photographed. This attenuation of light intensity protects the sample and detector from strong X-ray irradiation. The primary X-ray image is converted into a visible image on a thin scintillator crystal CdWO4 of 100 μm in thickness. X-ray images are captured using a CCD camera (Vieworks, VH-2MC). The field-of-view with a 10× objective lens attached in front of the camera is approximately 1.2 mm × 0.9 mm in physical dimension. The pixel size is about 0.74 μm.


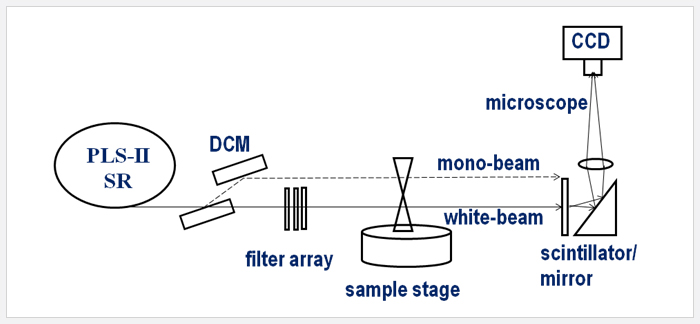


**Figure S6.** Experimental set-up for X-ray microscopy (XM) at PAL.

**Transport of permeate molecules (Pyrene or Rhodamine) through AuNP-PEG nanocomposites in aqueous solution.** The volume and height of the AuNP-PEG nanocomposites embedded with PVA matrix (M_w_~72,000 Merck, 3 wt %) are carefully designed as a pellet (0.1 cm diameter with 5 cm length). AuNP-PEG composite pellets are loaded in a thin-walled (0.01 mm) glass tube (Hampton Research, CA, USA). Humidity and temperature are controlled as illustrated in Figure 2a.

Rhodamine 6G is an ionic molecule with high water solubility (400 g/L). It exhibits dark orange color in water and is often used as an effective tracer for water flow. With high water solubility, rhodamine 6G is directly dissolved in water and used as a saturated solution. By contrast, pyrene is a nonionic hydrophobic molecules and is sensitive to fluorescence detection. A stock solution of pyrene (water solubility: 0.135 mg/L) is prepared by adding a known weight of the compound in 20 wt% ethanol in water. The mixture is sonicated to yield a clear solution. Pyrene solution (2 μM) is prepared by dilution wherein the ethanol concentration is 0.5%. Such a small concentration of the ethanol is used so the stimuli-responsiveness of AuNP-PEG nanocomposites is not affected.

The concentration-controlled permeate solutions are injected into the inlet line at a designed time interval (Micro-Liter OEM Syringe Pump Modules, Harvard Apparatus, MA, USA) (Figure 2a). A series of eluted permeates are measured and recorded using a spectrophotometer (2489 UV/Visible Detector, Waters, MA, USA). Empower 3 Chromatography Data Software is employed for data analysis. Spectra are obtained at a scan rate of 100 nm/s, and intensities are collected at an interval of 0.5 nm. For long-term and quick scanning, the wavelength is controlled to a narrow range. The emission and excitation wavelengths of 428 and 326 nm, respectively, with slit width of 2.50 nm, are used for both emission and excitation spectra of pyrene. The excitation wavelength is set at 326 nm because pyrene shows significant absorbance at this value. For rhodamine 6G spectra, the excitation wavelength of 485 nm, excitation slit width of 10 nm, and emission slit width of 2.50 nm are used. All spectra are obtained at 20 °C. To precisely control the local temperature of the designed nanocomposites, a multi-point temperature controller (MPC, Briskheat Corp., OH, USA) with resistance heating coils made of coiled nichrome or chromel wire (MOR Electric Heating Assoc. Inc., MI, USA) is employed.

**Table S1.** Experimental conditions and the fitting parameters for contractile action of a one-valve system.


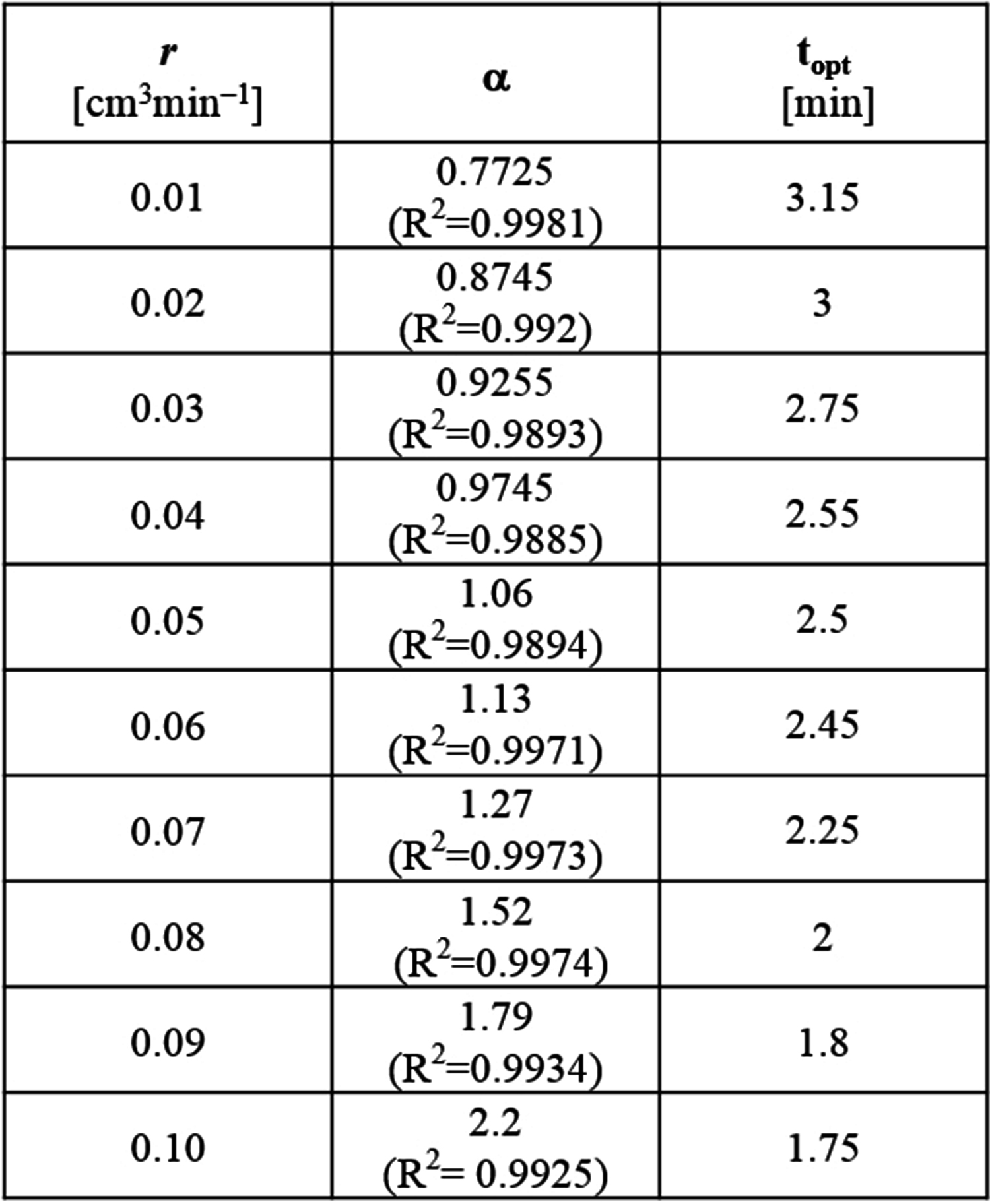


**Table S2.** Experimental conditions and the fitting parameters for sequential contractile actions of a multi-valve system.

**
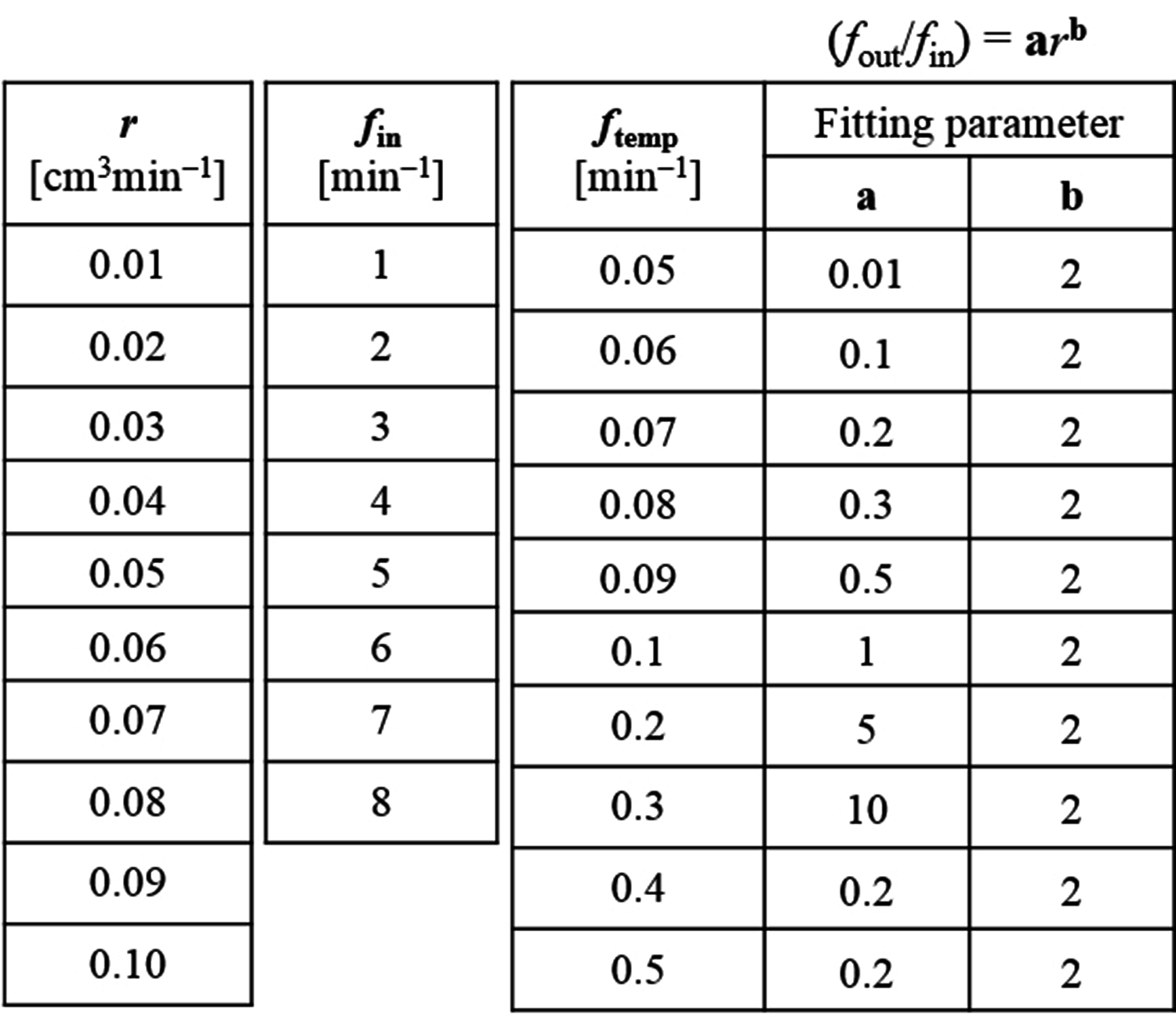
**
